# Hybrid ARQ Scheme with Autonomous Retransmission for Multicasting in Wireless Sensor Networks

**DOI:** 10.3390/s17030463

**Published:** 2017-02-25

**Authors:** Young-Ho Jung, Jihoon Choi

**Affiliations:** School of Electronics and Information Engineering, Korea Aerospace University, 200-1 Hwajeon-Dong, Goyang-si, Gyunggi-do 10540, Korea; jihoon@kau.ac.kr

**Keywords:** hybrid automatic repeat request (HARQ), autonomous retransmission, multicast service, feedback overhead, power saving

## Abstract

A new hybrid automatic repeat request (HARQ) scheme for multicast service for wireless sensor networks is proposed in this study. In the proposed algorithm, the HARQ operation is combined with an autonomous retransmission method that ensure a data packet is transmitted irrespective of whether or not the packet is successfully decoded at the receivers. The optimal number of autonomous retransmissions is determined to ensure maximum spectral efficiency, and a practical method that adjusts the number of autonomous retransmissions for realistic conditions is developed. Simulation results show that the proposed method achieves higher spectral efficiency than existing HARQ techniques.

## 1. Introduction

Multicast services have received considerable attention as a means for supporting point-to-multipoint applications such as video conferencing, mobile broadcasting, multimedia streaming services, and news distribution. These applications are achieved in mobile networks by the inclusion of an enhanced multimedia broadcast multicast service (eMBMS) and an enhanced multicast broadcast service (E-MBS) in current wireless communication standards such as the 3rd Generation Partnership Project Long Term Evolution (3GPP LTE) and IEEE 802.16m include an enhanced, respectively [1,2]. The original version of the eMBMS system was mainly focused on broadcast service based on single frequency network consisting of multiple cells [3] and did not support uplink feedback channels. Therefore, the modulation and coding scheme (MCS) of eMBMS packets need to as robust as possible so as to accommodate the user in the worst channel quality [4].

Recently, there has been a growing need for communication systems that efficiently provide multicast services rather than broadcast services in various applications such as public safety services, surveillance services, smart grid services, and reconnaissance services using unmanned air-vehicles (UAVs) [5,6,7,8,9]. In multicast services, it is important to securely and efficiently share information in a wireless sensor network (WSN) which is composed of various sensors such as cameras, thermal sensors, air pollution sensors, and smart meters. For example, if an effective cooperative search service that uses multiple UAVs equipped with cameras and thermal sensors, the information about search target, search area, and surrounding obstacles must be shared with all the UAVs. In a smart grid service, real-time power cost information must be reliably transmitted to all smart meters. Therefore, unlike a broadcasting service, multicasting in WSNs requires a guarantee of quality of service (QoS). In order to improve the spectral efficiency of multicast services, 3GPP Technical Specification Group Radio Access Network Working Group 2 has studied the Single-Cell Point-to-Multipoint (SC-PtM) transmission for LTE Release 13 [10]. In case of SC-PtM transmission, the need to support uplink feedback channels in link adaptation schemes has been studied and significant gain in spectral efficiency was observed in the adaptive MCS selection and hybrid automatic repeat request (ARQ) [11].

In ARQ schemes, the receiver examines a packet error using an error detection code such as cyclic redundancy check, and sends an acknowledgement (ACK) or no acknowledgement (NACK) message to the transmitter. In order to enhance link performance and reliability, ARQ schemes are combined with channel coding. This yields hybrid ARQ (HARQ) mechanisms, in which the receiver decodes a retransmitted packet in conjunction with previously transmitted erroneous packets. Recent results concerning HARQ are presented in [12] and the references therein. The HARQ schemes used in 3GPP LTE and IEEE 802.16m are described in [13,14,15], and the use of HARQ for multicasting has been studied in [5,16,17,18,19].

When the conventional HARQ method is applied to downlink multicast packets, multiple ACK channels are assigned in the uplink for multicast users. Consequently, the feedback overhead in the uplink is increased with an increasing number of multicast users. A proposal to reduce the ACK/NACK feedback overhead from user specific feedback involved a NACK based common feedback channel [18,19]. The physical resources for feedback are shared by all mobile stations (MSs) in the multicast group and the MSs that fail to decode send NACK signals. The NACK-based common feedback channel can save feedback overhead in the high signal to interference plus noise ratio (SINR) regime if multiple antennas are used, but the probability of misdetection is quite high in realistic conditions. Moreover, there exists a risk of ACK or NACK misinterpretation in the NACK-based common feedback channel [5,20]. Therefore, only the user specific feedback channel is considered for LTE SC-PtM transmission [10].

To reduce feedback overhead by means other than NACK-based common feedback, we propose a new HARQ scheme for transmitting multicast packets. In a conventional HARQ scheme, the receiver sends back an ACK or NACK signal in accordance with packet reception. By contrast, our proposed HARQ scheme performs autonomous retransmission at the beginning of packet transmission. That is, it automatically retransmits a packet a predetermined number of times without ACK/NACK feedback, and then performs the HARQ operation in the conventional HARQ method. Since the proposed method does not use ACK channels during autonomous retransmission, the feedback overhead is reduced. We define a spectral efficiency metric considering both the HARQ gain and the feedback overhead, and determine the optimal number of autonomous retransmissions that maximizes its value. We then propose a practical algorithm that adjusts the number of autonomous retransmissions for application in real communication systems. By way of computer simulations, the proposed scheme is compared with the conventional HARQ scheme in terms of spectral efficiency. As the number of MSs in the multicast group increases, the resource saving of the proposed HARQ scheme relative to the conventional HARQ scheme also increases. Therefore, the proposed scheme is potentially a practically applicable multicast transmission scheme in WSNs that can enhance spectral efficiency while guaranteeing QoS.

This paper is organized as follows. In Section 2, a brief introduction to the multicast transmission scheme using the conventional HARQ method is made. The proposed HARQ algorithm is explained in Section 3. In Section 4, we derive the optimal number of retransmissions for the proposed HARQ scheme based on a spectral efficiency metric in order to evaluate its performance as a component of multicast transmission. In Section 5, a practical algorithm adjusting the number of retransmissions is developed. Simulation results and conclusions are presented in Section 6 and Section 7, respectively.

## 2. Conventional HARQ Mechanism

**Figure 1:**
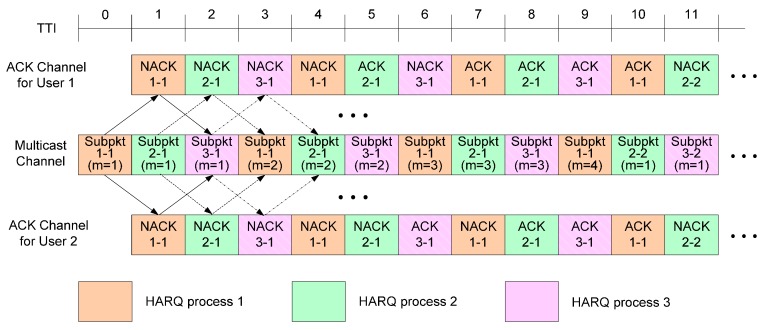
Conventional HARQ scheme. Subpkt *x*-*y* (*m* = *n*) means the *n*-th transmission of the *y*-th HARQ packet by the *x*-th HARQ process.

3GPP LTE and IEEE 802.16m adopted the multi-process stop-and-wait HARQ mechanism, which interlaces several independent stop-and-wait HARQ operations in time so as to fully utilize transmission resources [13,14,15]. Figure 1 describes the conventional HARQ scheme, where multicast packets are transmitted to two users by interlacing three HARQ processes, and the decoding delay is one transmit time interval (TTI) in both the uplink and downlink transmission. In this case, the transmitter repeatedly sends a packet until ACK messages are received from all the multicast users or until the limit for the number of retransmissions is reached. For example, in Figure 1, the transmitter starts to send a new packet when TTI = 10 after receiving ACK messages from both users when TTI = 9 (ACK messages for Subpkt 2-1 were transmitted when TTI = 8). Since the conventional HARQ method for multicast is a direct extension of the conventional HARQ scheme for unicast, it can be easily implemented in practical communication systems. However, the overhead from ACK/NACK feedback rapidly increases as the number of multicast users increases.

## 3. Proposed HARQ Algorithm

The MCS of multicast packets is determined by the user with the worst channel quality [16,17]. When HARQ is used, the MCS level is usually determined such that the worst user can achieve the target packet error rate (PER) within the maximum number of HARQ transmissions [18]. Under this scheme, the receiver achieves improved diversity gain, but the ACK feedback overhead increases as the number of retransmissions increase, as mentioned in Section 2.

**Figure 2:**
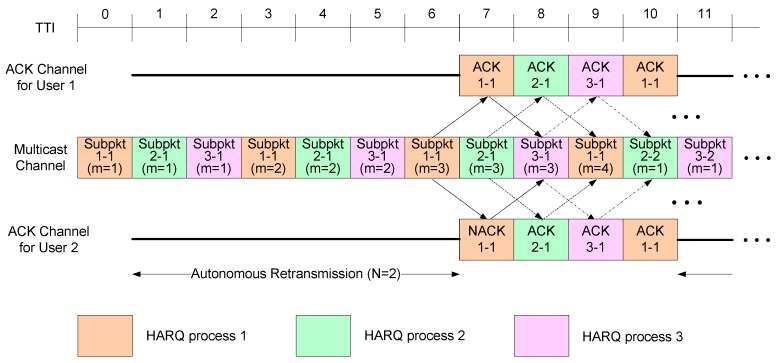
Proposed HARQ scheme when *N* = 2.

When the target number of HARQ transmissions is large—the target number of HARQ transmissions is determined by taking into account the packet transmission delay, the average throughput, and the buffer size at both transmitter and receiver—the resulting probability that a HARQ packet will be successfully decoded in the first or second transmission is row. Based on this observation, we propose a new HARQ scheme that automatically retransmits a data packet a predetermined number of times, N, without receiving ACK information. This process is referred to as ‘autonomous retransmission’ for the rest of this paper. Starting from the (N+1)-th transmission, the proposed algorithm operates in the same manner as the conventional HARQ scheme, where packet retransmission is determined based on ACK information received through the feedback channel. Figure 2 illustrates the proposed HARQ method when three HARQ processes are used to transmit to two multicast users, where N = 2. The users do not send ACK/NACK messages for the first and second packet transmissions, and start to send ACK information from the third transmission onwards. Consequently, ACK feedback overhead is reduced. Since a multicast transmission requires multiple ACK channels in the uplink for multicast users, the feedback overhead increases with an increase in the number of users. Using the proposed HARQ scheme, we can reduce the feedback overhead for multicast transmission, thereby increasing the uplink throughput. In Figure 2, users do not send ACK messages from TTI = 1 to TTI = 6. Consequently, other users can exploit these unused resources to transmit control signals and uplink data packets.

Let us define $M_{\max}$ as the maximum number of HARQ transmissions including initial transmission and retransmissions. The proposed scheme is identical to the conventional HARQ method when N = 0, but the packet is transmitted without HARQ when $N=M_{\max}-1$. Since the parameter N provides the tradeoff between the HARQ gain in the downlink and the feedback overhead in the uplink, it is important to determine the parameter N in order to optimize the overall efficiency of a communication link.

## 4. Analysis of Spectral Efficiency

To find the optimal parameter N, we derive a spectral efficiency metric by considering both the downlink and the uplink. The number of bits included in a HARQ packet is expressed as

(1) $$N_{B}=\alpha \beta R_{d},$$

where α is the modulation index, β is the effective code rate, and $R_{d}$ is the resource size for the multicast packet. For example, 100 symbols generated by quadrature phase shift keying (QPSK) and 1/2 channel code includes 100 bits. To transfer a packet using the HARQ operation, HARQ subpackets which contain the same information bits are repeatedly transmitted. Assuming that when a packet is being transmitted, the number of multicast users, K, to which it is being simultaneously transmitted using H-ARQ is constant, the total resource size required for both downlink and the uplink transmission is given by

(2) $$R_{t}=R_{d}L+KR_{u}Q,$$

where $R_{u}$ is the effective resource size needed to send an ACK message during uplink transmission, and L denotes the average number of HARQ transmissions required during downlink transmission to terminate a packet transfer, and Q denotes the average number of ACK transmissions required during uplink transmission to terminate a packet transfer. In the proposed HARQ method, Q is less than or equal to L, whereas Q is equal to L in the conventional HARQ scheme. Using (1) and (2), we define a spectral efficiency metric, η, as

(3) $$\begin{array}{ll} \eta & =(1-\gamma )\frac{N_{B}}{R_{t}},\\ & =\frac{(1-\gamma )\alpha \beta R_{d}}{R_{d}L+KR_{u}Q}, \end{array}$$

where γ is the target PER after the maximum number of HARQ transmissions has occurred. In (3), the spectral efficiency decreases as the number of multicast users decrease. This is due to the increased availability of resources for ACK feedback.

### 4.1. Spectral Efficiency of Conventional HARQ Scheme

Since Q is equal to L in the conventional scheme, its spectral efficiency metric, $\eta_{c}$, is expressed as

(4) $$\eta_{c}=\frac{(1-\gamma )\alpha \beta R_{d}}{(R_{d}+KR_{u})L_{c}},$$

where $L_{c}$ is the average number of HARQ transmissions per packet in the conventional method. In (4), all parameters except $L_{c}$ are determined by the scheduler and the physical specifications for packet transmission. Due to this, the transmitter stores all this information. Since $L_{c}$ is closely related to link performance, we derive an expression to compute $L_{c}$ using PER values in the HARQ link. If a random variable M is the number of transmissions required until a packet transfer is terminated, then, $L_{c}$ is given by

(5) $$L_{c}=\sum_{m=1}^{M_{\max}}m\cdot P[M=m]=\sum_{m=1}^{M_{\max}}m\cdot p_{c,m},$$

where *m* is the index of the HARQ transmission trial for a multicast packet, $p_{c,m}=P[M=m]$ denotes the probability that a packet transfer is terminated at the *m*-th transmission for the conventional HARQ. When M=1, all users successfully decode the packet at the first transmission. Thus, $p_{c,1}$ is computed as

(6) $$p_{c,1}=\prod_{k=1}^{K}\left(1-q_{1,k}\right),$$

where $q_{m,k}$ is the PER corresponding to the *m*-th HARQ transmission to the *k*-th user. In general, the receiver combines the retransmitted packet with previously transmitted erroneous packets to achieve the HARQ gain. Thus, $q_{m,k}$ decreases as *m* is increased. As with P[M≤1] in (6), P[M≤2] is denoted as

(7) $$P[M\leq 2]=\prod_{k=1}^{K}\left(1-q_{1,k}q_{2,k}\right).$$

From (6) and (7), we get

(8) $$\begin{array}{ll} p_{c,2} & =P[M\leq 2]-p_{c,1}\\ & =\prod_{k=1}^{K}\left(1-q_{1,k}q_{2,k}\right)-p_{c,1}. \end{array}$$

By generalizing the procedure shown in (6)–(8), $p_{c,m}$ can be expressed as

(9) $$p_{c,m}=\prod_{k=1}^{K}\left(1-\prod_{n=1}^{m}q_{n,k}\right)-\sum_{n=1}^{m-1}p_{c,n},$$

where $1\leq m\leq M_{\max}-1$. When the maximum number of transmissions is reached, packet transfer is terminated without considering whether the transmission was a success or failure. Therefore, $P[M=M_{\max}]$ is given by

(10) $$p_{c,M_{\max}}=1-\sum_{m=1}^{M_{\max}-1}p_{c,m}.$$

Note that Equation (10) is obtained from the fact that $p_{c,1}+p_{c,2}+\cdots +p_{c,M_{\max}}=1$. From (9) and (10), $p_{c,m}$ is expressed as

(11) $$p_{c,m}=\{\begin{array}{cc} \prod_{k=1}^{K}\left(1-\prod_{n=1}^{m}q_{n,k}\right)-\sum_{n=1}^{m-1}p_{c,n}, & \mathrm{for}\;1\leq m\leq M_{\max}-1,\\ 1-\sum_{n=1}^{M_{\max}-1}p_{c,n}, & \mathrm{for}\;m=M_{\max}. \end{array}$$

When the value of $q_{m,k}$ is known, $p_{c,m}$ can be computed using (11), and $\eta_{c}$ and $L_{c}$ can, in turn, be evaluated by (4) and (5), respectively.

### 4.2. Spectral Efficiency of Proposed HARQ Scheme

Since N autonomous retransmissions are performed in the proposed scheme, a packet is repeatedly transmitted at least (N+1) times. Therefore, if we define $p_{p,m}=P[M=m]$ as the probability that a packet transfer is terminated at the *m*-th transmission for the proposed HARQ, $p_{p,m}=0$ for m≤N and $p_{p,N+1}$ is expressed as

(12) $$\begin{array}{ll} p_{p,N+1} & =\prod_{k=1}^{K}\left(1-\prod_{n=1}^{N+1}q_{n,k}\right)\\ & =P[M\leq N+1]\\ & =\sum_{m=1}^{N+1}p_{c,n} \end{array}.$$

Because $\sum_{n=1}^{N+1}p_{p,n}=\sum_{n=1}^{N+1}p_{c,n}$ and there is no difference between the conventional HARQ and the proposed HARQ for m>N+1, $p_{p,m}$ can be expressed as

(13) $$p_{p,m}=p_{c,m},$$

for $N+1<m\leq M_{\max}$. So, the average number of HARQ transmissions per packet, $L_{p}(N)$, is denoted as a function of N, as

(14) $$L_{p}(N)=\sum_{m=N+1}^{M_{\max}}m\cdot p_{p,m}.$$

Since users do not send ACK messages for the first N transmissions, the average number of ACK transmissions per packet, $Q_{p}(N)$, is given by

(15) $$Q_{p}(N)=L_{p}(N)-N.$$

By substituting (14) and (15) in (3), the spectral efficiency metric for the proposed algorithm, $\eta_{p}(N)$, is expressed as

(16) $$\eta_{p}(N)=\frac{(1-\gamma )\alpha \beta R_{d}}{(R_{d}+KR_{u})L_{p}(N)-KR_{u}N}.$$

When the value of $q_{m,k}$ is known, $p_{p,m}$ can be computed using (11)–(13), and $L_{p}(N)$ and $\eta_{p}(N)$ can, in turn, be evaluated by (14) and (16). By calculating the spectral efficiency metric for all possible value of N, the optimal N, $N^{*}$, and the corresponding maximum spectral efficiency metric, $\eta_{p,\max}$, are computed as

(17) $$N^{*}=\underset{N}{\arg}\underset{0\leq N\leq M_{\max}-1}{\max}[\eta_{p}(N)],$$

and

(18) $$\eta_{p,\max}=\underset{0\leq N\leq M_{\max}-1}{\max}[\eta_{p}(N)].$$

In (17), the optimal number of autonomous retransmissions is determined as the value corresponding to $\eta_{p,\max}$. Equations (17) and (18) theoretically provide the $N^{*}$ and the corresponding maximum spectral efficiency, given multicast environments with HARQ operation. In practical systems, however, it is difficult to find the $N^{*}$ and the corresponding $\eta_{p,\max}$, because the computation of $\eta_{p}(N)$ in (16) requires the knowledge of PER curves for the physical link depending on time-varying channel conditions.

## 5. Practical Implementation of Proposed HARQ Scheme

For practical implementation, we propose an adaptive algorithm that adjusts the number of autonomous retransmissions blockwise, such that the value N is fixed during an update interval and can be changed between update intervals. Specifically, the proposed algorithm adjusts the value of N used for the current update interval by using the estimated $\left\{p_{p,m};1\leq m\leq M_{\max}\right\}$ from the previous update interval. To adaptively adjust the number of autonomous retransmissions, we need two criteria—one is used to increase the value of N and the other is used to decrease the value of N. To derive the criterion used to increase N, we compare $\eta_{p}(N)$ and $\eta_{p}(N+1)$. In other words, the value N is increased, when $\eta_{p}(N+1)$ is greater than $\eta_{p}(N)$. Since the numerators of $\eta_{p}(N)$ and $\eta_{p}(N+1)$ are the same, we compute the difference between the denominators of $\eta_{p}(N)$ and $\eta_{p}(N+1)$. For $\eta_{p}(N)$, the denominator of (14) is defined by

(19) $$R_{t}(N)=(R_{d}+KR_{u})L_{p}(N)-KR_{u}N.$$

The difference between the denominators of $\eta_{p}(N)$ and $\eta_{p}(N+1)$ is written as

(20) $$\begin{array}{ll} d(N,N+1) & =R_{t}(N)-R_{t}(N+1)\\ & =(R_{d}+KR_{u})[L_{p}(N)-L_{p}(N+1)]+KR_{u} \end{array}.$$

Using (12), d(N,N+1) is computed as

(21) $$d(N,N+1)=KR_{u}-(R_{d}+KR_{u})p_{p,N+1}.$$

Since $p_{p,N+1}=P[M\leq N+1]$ is the probability that a packet transfer is terminated by the (N+1)−th transmission, the estimate can be easily calculated from the transmission history of previous HARQ packets. In (21), d(N,N+1)>0 implies that $\eta_{p}(N)<\eta_{p}(N+1)$. Thus, the current value of N increases when d(N,N+1)>0. As a next step, we need to determine the criterion that can be used to decrease the value of N. As in (19–21), we can derive d(N−1,N) as follows

(22) $$d(N-1,N)=KR_{u}-(R_{d}+KR_{u})P[M\leq N].$$

In this case, P[M≤N] is not available for the HARQ process with N autonomous retransmissions, because a packet transfer is not completed before the (N+1)-th transmission. Therefore, d(N−1,N) cannot be computed in the proposed HARQ scheme with N autonomous retransmissions. Thus, we propose an alternative approach to compute d(N−1,N). When the number of autonomous retransmissions remains unchanged during a predetermined time interval, we decrease the number of autonomous retransmissions. Then, it is possible to compare $\eta_{p}(N-1)$ and $\eta_{p}(N)$using (22). In the proposed HARQ scheme, the procedure for adjusting the number of autonomous retransmissions is summarized as follows

1. Initialization: N = 0.
2. Estimation of $p_{p,N+1}$ during the current update interval.
3. Computation of d(N,N+1): use (21).
4. Adjustment of N:
  If d(N,N+1)>0, then $N=\min(N+1,M_{\max}-1)$.
  Else if N is not changed for T consecutive update intervals, then N=max(N−1,0).
5. Repeat steps 2–4 for the next update interval.

In step 5, T is a positive integer which is determined based on a tradeoff between the adaptation speed and the steady-state performance. The steady-state performance for various T values is provided in Section 6.

## 6. Simulation Results

In this section, the performance of the proposed HARQ scheme is compared with that of the conventional HARQ scheme through system level simulations (SLSs) for multicast transmission in the IEEE 802.16e downlink [21]. Although 3GPP LTE is more popular than IEEE 802.16e system, since 3GPP LTE has more complex structure of HARQ operation, IEEE 802.16e is easier to apply the proposed autonomous retransmission without changing the current standard than LTE. So, we chose IEEE 802.16e for performance evaluation. Note that the proposed autonomous retransmission-based HARQ transmission can be applied to various future standards for wireless multicast. We used MATLAB for simulator development. To generate time-correlated fading channels, the ITU-R Pedestrian B channel with 10 km/h velocity was used [22]; the carrier frequency was 2.5 GHz; the bandwidth was 10 MHz with 1024 subcarriers; the sampling rate was 11.2 MHz; and the frame duration was 5 ms. For a HARQ operation, multicast packets with 384 bits were transmitted to the downlink partial use of subcarriers (PUSC) zone; $R_{u}$ was set to 24 (a half slot in the uplink); $M_{\max}$ was 4; four independent HARQ processes were used; the convolutional turbo code (CTC) with a code rate of 1/2 was used; Chase combining was employed at the receivers; update interval N for the proposed method was set to 100 frames; and the downlink SINR was assumed to be available to the transmitter through feedback. For random packet error generation, we used MCS levels in Table 1 and the downlink PER curves shown in [23]. Each point of the simulation results was obtained by averaging the spectral efficiency metrics over more than 200 random drops of mobile users and corresponding fading channels.

**Figure 3:**
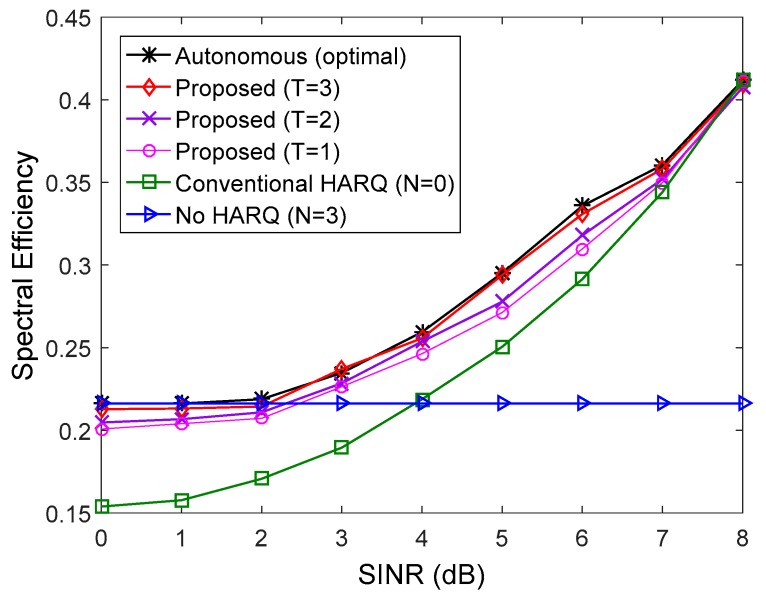
Comparison of spectral efficiency when *K* = 10, MCS level is 6, and all users have the same downlink SINR.

**Figure 4:**
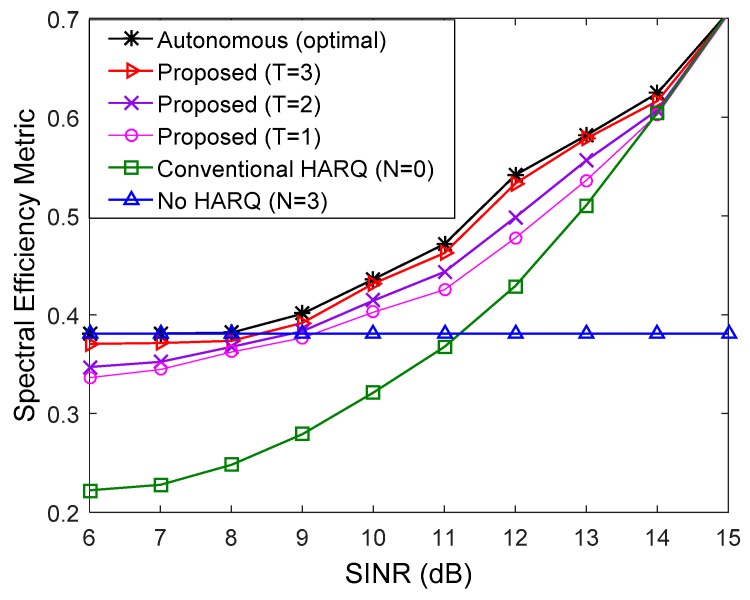
Comparison of spectral efficiency when *K* = 10, MCS level is 8, and all users have the same downlink SINR.

**Figure 5:**
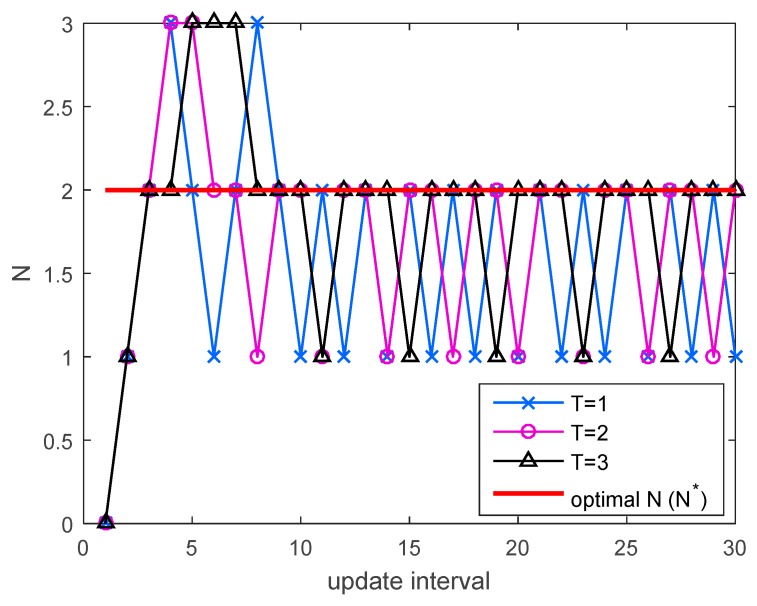
An example of adaptation of *N* for different value of *T* in the simulation environments of Figure 4 at SINR = 11 dB.

In Figure 3 and Figure 4, the spectral efficiency of the proposed method is compared to that of the conventional method when the number of multicast users is 10 (K=10) and all users have the same downlink average SINR. We used QPSK and 16 quadrature amplitude modulation (16QAM) in Figure 3 and Figure 4, respectively. For comparison, the spectral efficiency of transmission without the HARQ scheme (N=3) was also presented. In the low SINR regime, the number of autonomous retransmissions for the proposed HARQ method is increased to reduce feedback overhead resulting from high PER. Thus, the proposed method performs better than the conventional HARQ scheme. Since the number of autonomous retransmissions for the proposed method is decreased to avoid unnecessary autonomous retransmission in the high SINR regime, the proposed scheme achieves higher HARQ gain than the non-HARQ approach. The performance of the proposed scheme improves with increase in the update interval, T. When T=3, the proposed method performs very similarly to the autonomous method with the optimal N determined by (17), which is the theoretical upper limit. If the exact value of PER for all users in the multicast group is known at the transmitter, the optimal N can be determined by (17), but it is not possible in real situations due to the continuous change of channel conditions of multiple users. Therefore, the number of autonomous retransmissions, N is calculated using the procedure in Section 5. Figure 5 shows an example of adaptation of N with the different value of T in the simulation environment of Figure 4 at *SINR* = 11 dB. The optimal N calculated from (17) is two, and when T=3, the difference between the estimated N and the optimal N is minimized.

**Figure 6:**
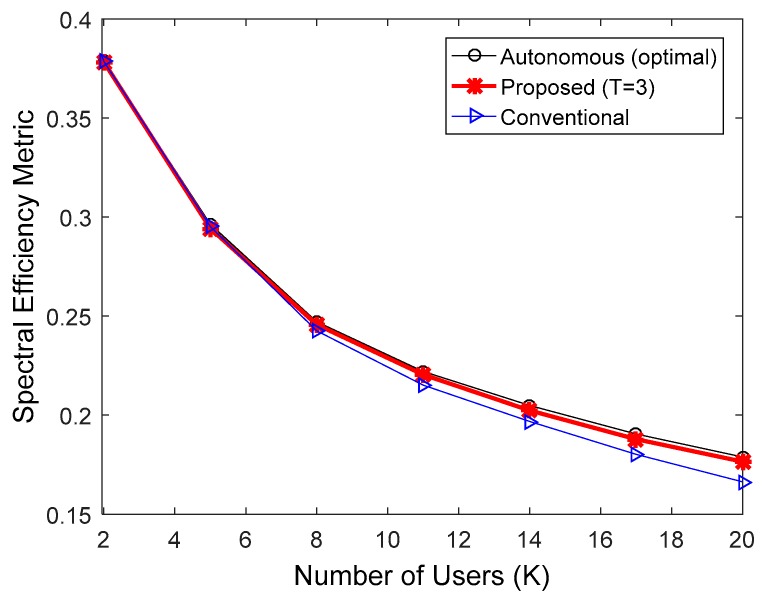
Comparison of spectral efficiency in the IEEE 802.16e downlink when the MCS level was fixed at 4.

**Figure 7:**
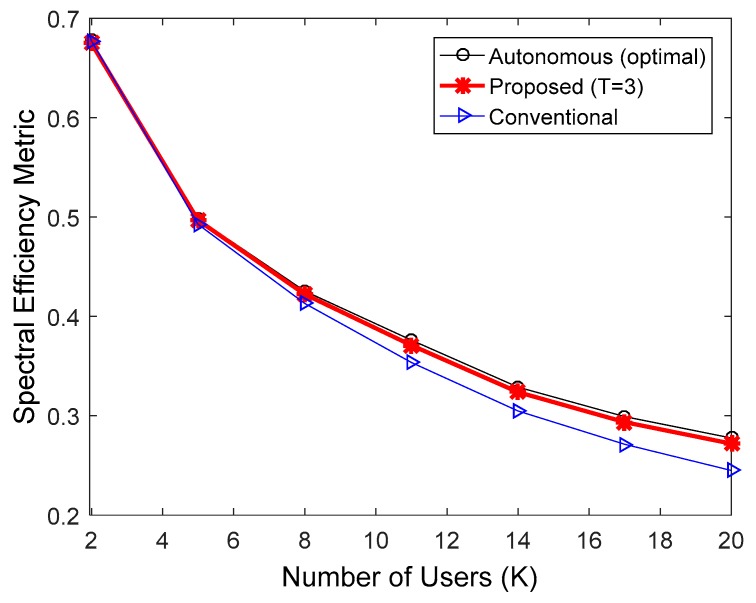
Comparison of spectral efficiency in the IEEE 802.16e downlink when the MCS level was fixed at 6.

**Figure 8:**
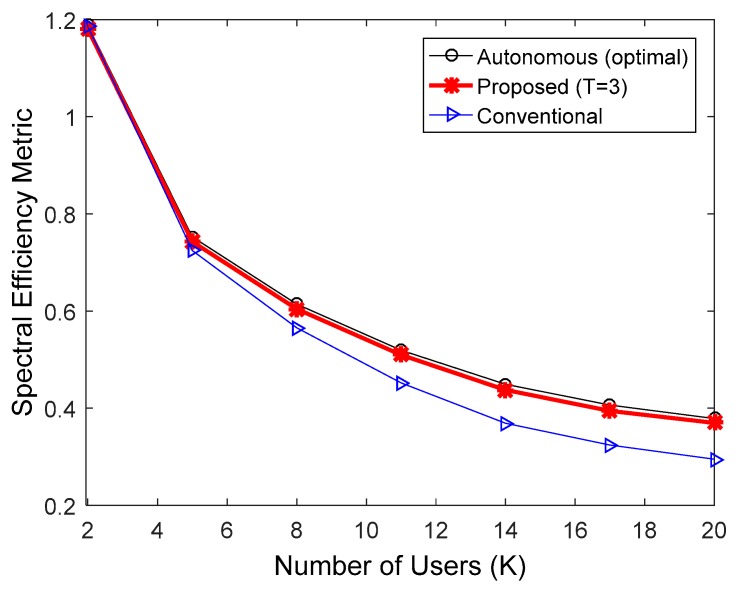
Comparison of spectral efficiency in the IEEE 802.16e downlink when the MCS level was fixed at 8.

Figure 6, Figure 7 and Figure 8 indicate the performance of the proposed HARQ technique in the IEEE 802.16e downlink, when multicast users were randomly dropped within the coverage of a base station. To take into account the practical IEEE 802.16e communication environments, we performed the simulations in accordance with Section 2.4 of [24]. In SLS, the following parameters were used: 19 cells with 1 km radius; one antenna each at the transmitter and receiver sides; ITU-R path loss model for vehicular environment [22]; log-normal shadowing with a standard deviation of 8.9 dB and a cell-to-cell correlation coefficient of 0.5; and antenna height set to 10 m at the transmitter and 1.5 m at the receiver. Figure 6, Figure 7 and Figure 8 compare spectral efficiencies when the MCS level was fixed at 4, 6, and 8, respectively. Given an MCS level, the multicast users were dropped so that the user with the worst downlink SINR had a PER less than 0.01. Furthermore, T = 3 was used for the proposed HARQ method considering the results in Figure 3 and Figure 4. The proposed algorithm requires less ACK channel overhead than the conventional HARQ scheme. Therefore, the proposed algorithm exhibited better spectral efficiency than the conventional scheme. The performance of the proposed scheme relative to the conventional H-ARQ scheme increases with an increasing number of users, because the feedback overhead is proportional to the number of users. In addition, the feedback overhead is more influential when the modulation order product code rate (MPR) is high. Consequently, the proposed scheme achieved the highest spectral efficiency gain when MCS level 8 was used. As before, the performance of the proposed method was very similar to that of the autonomous method with optimal N.

**Figure 9:**
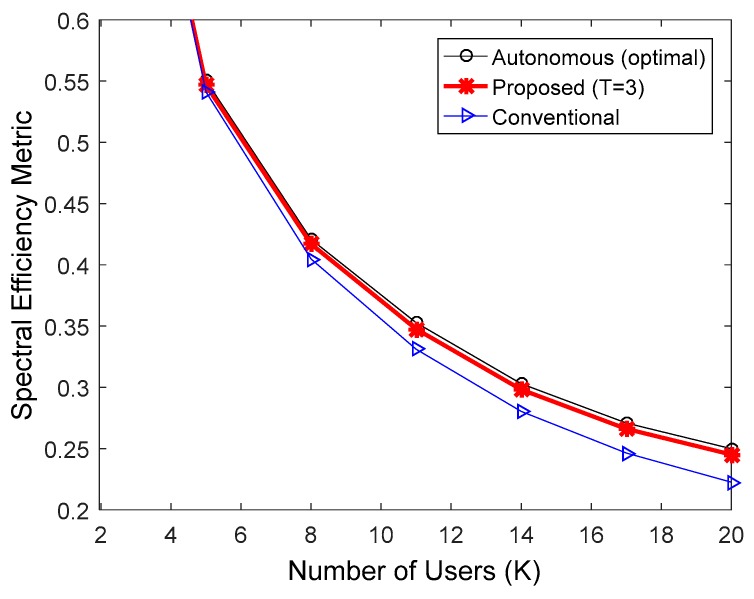
Comparison of spectral efficiency in the IEEE 802.16e downlink when MCS levels were dynamically changed over 4–11.

Figure 9 shows spectral efficiency in the IEEE 802.16e downlink, obtained by SLS in accordance with Section 2.4 of [24]. The simulation was performed in a similar manner to the SLSs in Figure 6, Figure 7 and Figure 8, except that the MCS levels were dynamically changed over 4–11 based on the downlink SINRs of multicast users. The MCS level of each drop was determined by considering the user with the worst SINR, so that the PER for multicast packets was less than 0.01. As before, the proposed method with T = 3 was used. The proposed algorithm presented better spectral efficiency than the conventional method, because it provides good tradeoff between the HARQ gain and the feedback overhead by properly adjusting the value N. Since the feedback overhead is proportional to K, the spectral efficiency gain increased with an increasing number of users. Overall, the spectral efficiency of the proposed algorithm was very similar to that of the autonomous method with theoretically optimal N.

**Figure 10:**
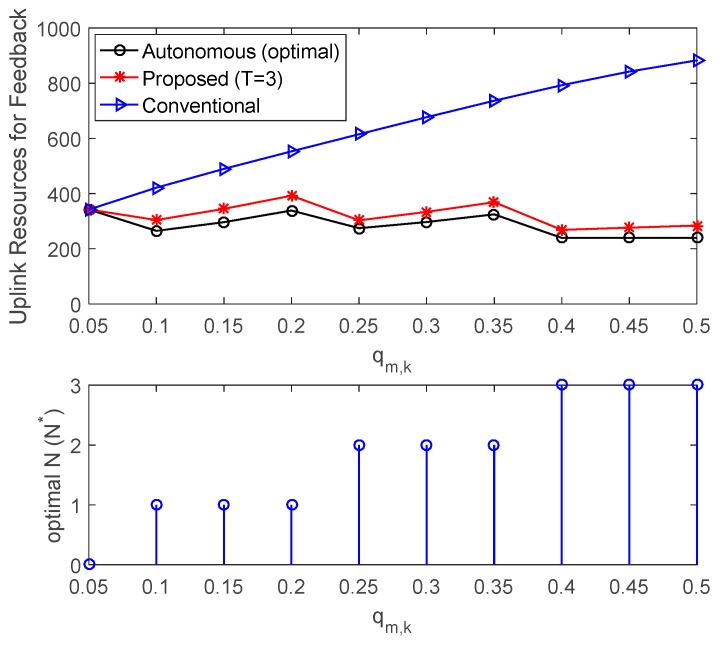
Comparison of required uplink resources for ACK/NACK feedback and optimal *N* according to different value of *q_m,k_* when *K* = 10.

Another advantage of the proposed HARQ is power saving of the sensor nodes. The transmission power of the MSs is proportional to the amount of uplink resources for feedback. Figure 10 compares the sum of required uplink resource for ACK/NACK feedback and optimal N for different values of $q_{m,k}$ when K=10. It is assumed that all users have the same $q_{m,k}$ for all HARQ transmissions. In the conventional HARQ, since the average number of ACK/NACK transmissions increases as $q_{m,k}$ increases, the uplink feedback resources increase in proportion to the increase of $q_{m,k}$. However, since the number of autonomous transmissions also increases as $q_{m,k}$ increases in the proposed HARQ, the required uplink feedback resources are maintained at almost the same level regardless of $q_{m,k}$. Therefore, the proposed HARQ can save more uplink transmission power as $q_{m,k}$ increases. For example, when $q_{m,k}=0.1$, the proposed HARQ with T=3 can save 27.8% of uplink transmission power for feedback as compared to the conventional HARQ, and when $q_{m,k}=0.3$ the proposed HARQ can save 50.7% of uplink transmission power.

## 7. Conclusions

In this paper, a new HARQ scheme with autonomous retransmission was proposed for use in multicast service. By providing a suitable tradeoff between the HARQ gain and the feedback overhead reduction, the proposed algorithm exhibited greater spectral efficiency than the conventional HARQ scheme. As a means to optimize the number of autonomous retransmissions, we introduced a spectral efficiency metric considering both the downlink and uplink, and derived a practical algorithm to adjust the number of autonomous retransmissions. When the importance of the downlink resource is different from that of the uplink resource, the proposed algorithm can be employed with a slight modification of the spectral efficiency metric. The proposed algorithm can be applied to communication standards for wireless multicast services such as SC-PtM of 3GPP and other future communication standards for WSN.

## Figures and Tables

**Table 1:** MCS levels.

MCS Level	Modulation	Code Rate	Spectral Efficiency (Bits/Symbol)
1	QPSK	1/12	0.167
2	QPSK	1/8	0.250
3	QPSK	1/6	0.333
4	QPSK	1/4	0.500
5	QPSK	1/3	0.667
6	QPSK	1/2	1.000
7	QPSK	2/3	1.333
8	16QAM	2/5	1.600
9	16QAM	1/2	2.000
10	16QAM	2/3	2.667
11	64QAM	2/3	4.000
